# Efficacy of sodium hypochlorite in overcoming antimicrobial resistance and eradicating biofilms in clinical pathogens from pressure ulcers

**DOI:** 10.3389/fmicb.2024.1432883

**Published:** 2024-07-10

**Authors:** Giorgia Fabrizio, Francesca Sivori, Ilaria Cavallo, Mauro Truglio, Luigi Toma, Francesca Sperati, Massimo Francalancia, Francisco Obregon, Luisa Pamparau, Daniela Kovacs, Fulvia Pimpinelli, Enea Gino Di Domenico

**Affiliations:** ^1^Department of Biology and Biotechnology "C. Darwin", Sapienza University of Rome, Rome, Italy; ^2^Microbiology and Virology, San Gallicano Dermatological Institute, IRCCS, Rome, Italy; ^3^Medical Directorate, IRCCS Regina Elena National Cancer Institute, Rome, Italy; ^4^UOSD Clinical Trial Center, Biostatistics and Bioinformatics, IRCCS San Gallicano Dermatological Institute, Rome, Italy; ^5^Cutaneous Physiopathology, San Gallicano Dermatological Institute, IRCCS, Rome, Italy

**Keywords:** NaOCl, biofilm, *Acinetobacter baumannii*, *Klebsiella pneumoniae*, *Staphylococcus aureus*, *Candida albicans*, sodium hypochlorite solution, skin, ESKAPE

## Abstract

Sodium hypochlorite (NaOCl) is widely recognized for its broad-spectrum antimicrobial efficacy in skin wound care. This study investigates the effectiveness of NaOCl against a range of bacterial and fungal isolates from pressure ulcer (PU) patients.

We analyzed 20 bacterial isolates from PU patients, comprising carbapenem-resistant *Klebsiella pneumoniae* (CRKP), multidrug-resistant Acinetobacter baumannii (MDRAB), methicillin-resistant *Staphylococcus aureus* (MRSA), methicillin-susceptible *Staphylococcus aureus* (MSSA), along with 5 *Candida albicans* isolates. Antibiotic resistance profiles were determined using standard susceptibility testing. Whole-genome sequencing (WGS) was employed to identify antimicrobial resistance genes (ARGs) and disinfectant resistance genes (DRGs). Genetic determinants of biofilm formation were also assessed. The antimicrobial activity of NaOCl was evaluated by determining the minimum inhibitory concentration (MIC) and the minimal biofilm eradication concentration (MBEC) for both planktonic and biofilm-associated cells.

CRKP and MDRAB showed resistance to fluoroquinolones and carbapenems, while MRSA exhibited resistance to β-lactams and levofloxacin. MSSA displayed a comparatively lower resistance profile. WGS identified significant numbers of ARGs in CRKP and MDRAB, with fewer DRGs compared to MRSA and MSSA. All isolates possessed genes associated with fimbriae production and adhesion, correlating with pronounced biofilm biomass production. NaOCl demonstrated substantial antimicrobial activity against both planktonic cells and biofilms. The MIC_90_ for planktonic bacterial cells was 0.125 mg/mL, and the MBEC_90_ ranged from 0.225 to 0.5 mg/mL. For planktonic C. *albicans*, the MIC_90_ was 0.150 mg/mL, and the MBEC_90_ was 0.250 mg/mL.

These results highlight the challenge in treating biofilm-associated infections and underscore the potential of NaOCl as a robust antimicrobial agent against difficult-to-treat biofilm infections at concentrations lower than those typically found in commercial disinfectants.

## 1 Introduction

Pressure ulcers (PUs) represent a global problem in hospitalized patients and a significant burden to healthcare systems. Recognized as largely preventable, PUs are integral to hospital safety protocols, aiming to enhance patient quality of life and reduce hospitalization durations (Kottner et al., 2019). Globally, approximately 12% of adult hospitalized patients experience pressure injuries, with the prevalence of those acquired in hospitals ranging from 7 to 9% (Li et al., 2020; Wang et al., 2024). Most PUs fail to progress beyond the inflammatory phase, and infection often impacts wound healing (Goldberg and Diegelmann, 2020). PUs are predisposed to microbial colonization and at high risk for multidrug-resistant (MDR) organism infections (Norman et al., 2016; Di Domenico et al., 2017; Binsuwaidan et al., 2023). Among MDR pathogens, ESKAPE (*Enterococcus faecium*, *Staphylococcus aureus*, *Klebsiella pneumoniae*, *Acinetobacter baumannii*, *Pseudomonas aeruginosa*, *Enterobacter* sp.) pathogens have caused a significant concern due to their resistance against antibiotics (Vale De Macedo et al., 2021). Complications related to treating infected PUs are not limited to the presence of MDR organisms. Increasing evidence suggests biofilm accounts for a major virulence factor in ulcers’ pathogenesis (Cavallo et al., 2024). Indeed, PUs offer an ideal environment for biofilm formation due to the reduced host immune response and the presence of necrotic tissues that promote microbial adhesion (Siddiqui and Bernstein, 2010; Zhao et al., 2013; Cavallo et al., 2024). Bacteria embedded in a mature biofilm matrix are highly tolerant to conventional antimicrobial treatments. As a result, biofilm bacteria are difficult to eradicate since they require a minimum inhibitory concentration (MIC) impossible to reach *in vivo* due to the side effects and the toxicity of the drugs at renal and/or hepatic levels (Percival et al., 2015; Di Domenico et al., 2022). In response to the escalating challenge of antibiotic resistance, which is often exacerbated by indiscriminate systemic therapy, the British Society for Antimicrobial Chemotherapy (BSAC) and the European Wound Management Association (EWMA) recommend prioritizing the use of non-antibiotic antimicrobials for treating infected wounds (Lipsky et al., 2016). Topical antiseptics are preferred over topical antibiotics because they are broader in their spectrum of activity and less likely to cause allergic reactions. The ideal antiseptic for managing an infected skin wound should combine therapeutic efficacy with tissue tolerability (Kramer et al., 2018). Sodium hypochlorite (NaOCl) solutions are considered among the first choices for skin wound care due to their effectiveness against a wide range of planktonic and biofilm microorganisms, their role in promoting physiological tissue repair, and their anti-inflammatory actions (Ciccia et al., 2018; Lineback et al., 2018; Myltykbayeva et al., 2020). The NaOCl has longed to be used as an antiseptic for patients on dialysis and to irrigate wounds and burns (Bruch, 2006; Cruz et al., 2006; Alvarez et al., 2010). The NaOCl is a cheaper antiseptic than chlorhexidine, slightly volatile and less flammable than alcohol, less vulnerable to deactivation by blood and serum protein than povidone-iodine, and less toxic than povidone-iodine (Alvarez et al., 2010). Besides, NaOCl has never been correlated with bacterial contamination and hospital outbreaks. The NaOCl has a broad spectrum of action and is an isotonic and physiological solution that does not cause any osmotic stress toward damaged tissues (Mourad and Hobro, 2020). Furthermore, due to the positive activity/tolerability ratio, the 0.05% NaOCl solution has to be considered one of the reference solutions for the disinfection of damaged skin (Maniscalco et al., 2023).

The present study was designed to comprehensively investigate the antimicrobial resistance patterns and biofilm production capabilities of ESKAPE pathogens and *Candida albicans* isolates derived from PUs. Specifically, it assessed the efficacy of NaOCl in combating these pathogens, focusing on its role against planktonic cells and disrupting biofilm structures. The findings are intended to contribute to a deeper understanding of biofilm resilience mechanisms and inform more effective treatment approaches for managing infections associated with PUs.

## 2 Materials and methods

### 2.1 Microbial strain collection

From the Microbial Strain Repository of the Laboratory of Microbiology and Virology, San Gallicano Dermatological Institute, Rome, a total of 25 consecutive bacterial isolates, including five carbapenem-resistant *K. pneumoniae* (CRKP), five multidrug-resistant *A. baumannii* (MDRAB), five methicillin-resistant (MRSA) and five methicillin-susceptible *S. aureus* (MSSA), and five *C. albicans* from patients with infected PUs admitted between August 2022 and July 2023 were included in the study. Antimicrobial susceptibility testing was assessed by the BD PhoenixTM automated microbiology system (Becton Dickinson Diagnostic Systems, Sparks, MD, United States). Multidrug-resistant (MDR) bacteria were defined according to the European Centre for Disease Prevention and Control (ECDC) criteria.^1^ The antifungal susceptibility testing was performed utilizing Sensititre YeastOne YO10 AST Plate (Thermo Fisher Scientific, United States) and following manufacturers’ instructions.

PUs were identified as localized skin and underlying tissue lesions caused by compression between bony prominences and external surfaces. When a patient presented with multiple ulcers, all ulcers exhibiting local signs of infection were cultured, and the stage of the most severe ulcer was assigned to the case. Infected PUs were categorized based on specific guidelines (Livesley and Chow, 2002). Ethical approval was secured from the Central Ethics Committee I.R.C.C.S. Lazio, Rome (Protocol 8,993—05.07.2022, trial registry number 1733/22), adhering to the Helsinki Declaration.

### 2.2 Antimicrobial susceptibility test of different concentrations of sodium hypochlorite

The minimum inhibitory concentration (MIC) of NaOCl against the selected pathogens was established through plate counting to determine the CFU/ml. A standard inoculum of 5 × 10^5^ colony-forming units (CFU)/mL was utilized. NaOCl was diluted in serial two-fold increments in cation-adjusted Mueller–Hinton broth (MHB). The MIC_90_ was identified as the lowest antibiotic concentration, resulting in a 90% reduction in bacterial survival compared to the untreated control (Sivori et al., 2022). All experiments were performed in triplicate.

### 2.3 Multiple antibiotic resistance index calculation

The multiple antibiotic resistance (MAR) index for each isolate was determined using the following equation:

MARindex=a/b,

Where a represents the number of antibiotics that the isolate resists, and b denotes the total number of antibiotics tested against the isolate (Krumperman, 1983).

### 2.4 Whole-genome analysis

Whole-genome sequencing (WGS) was performed on all bacterial strains included in this study. DNA for whole-genome sequencing (WGS) was extracted using QIAsymphony DSP Virus/Pathogen Kits (Qiagen, Hilden, Germany) following the manufacturer’s protocols. The quality of the DNA reads was enhanced by trimming using FastP v0.23.4, and the sequences were assembled with SPAdes v3.15.5 (Bankevich et al., 2012; Chen et al., 2018). These sequences were annotated using Prokka v1.14.6. A pan-genome analysis was conducted using Roary v3.13.0 (Page et al., 2015). Predictions for antibiotic resistance genes were performed utilizing the Comprehensive Antibiotic Resistance Database (CARD) v3.2.8 and the Resistance Gene Identifier (RGI) tool, specifically focusing on “perfect” and “strict” matches to high-quality reference sequences, with a threshold of 97% identity for inclusion (Alcock et al., 2023). Virulence factors were determined by using Blastn against the Virulence Factor Database (VFDB), requiring at least 80% coverage and 90% identity (Chen et al., 2016; Cavallo et al., 2022). The abundance of Clusters of Orthologous Groups (COG) categories was assessed using eggNOG-mapper v2.1.12 aligned with the eggNOG database v5.0, under default settings (Cantalapiedra et al., 2021).

### 2.5 Biofilm formation

Biofilm formation was quantified using crystal violet (CV) to assess biomass 24 h post-incubation. Sterile 96-well polystyrene plates were inoculated with 200 μL of a bacterial or yeast cell suspension (10^5^ CFU/mL) in MHB and incubated at 37°C for 24 h without agitation. Following incubation, the medium was discarded, and the wells were washed three times with 200 μL of sterile distilled water. The plates were then air-dried for 45 min before the adherent cells were stained with 200 μL of 0.1% CV solution for 15 min. Excess stain was removed by washing the wells three times with 200 μL of sterile distilled water. The stain absorbed by the biofilm-forming cells was solubilized in 200 μL of an ethanol-acetone mixture (4:1 ratio), and the absorbance of each well was measured at 570 nm (OD570) using a Multiskan SkyHigh spectrophotometer (Thermo Fisher Scientific, Ohio, United States). All experiments were conducted in triplicate and repeated independently at least three times (Di Domenico et al., 2018; Truglio et al., 2024).

### 2.6 Quantification of biofilm-forming cells

Cell suspensions diluted to approximately 5 × 10^5^ CFU/mL were used to inoculate a 96-well polystyrene flat-bottom plate with 100 μL of MHB for biofilm cultivation. After a 24-h incubation at 37°C, the wells were washed twice with sterile deionized water and resuspended in 100 μL of MHB. The biofilms were then thoroughly scraped, and viable cells were quantified by serial dilution and plating on blood agar for bacterial cultures or Sabouraud’s dextrose agar for *C. albicans* to determine CFU/ml.

### 2.7 Minimum biofilm eradication concentration (MBEC) assays of different concentrations of sodium hypochlorite

Cell suspensions, diluted to approximately 5 × 10^5^ CFU/mL, were utilized to inoculate a 96-well polystyrene flat-bottom plate with 100 μL of MHB for biofilm cultivation. After incubation at 37°C for 24 h, the wells were rinsed with 0.45% saline solution to eliminate nonadherent cells. Subsequently, the cells were resuspended in 100 μL of MHB containing serial dilutions of NaOCl. The plate was then incubated for an additional 20 h at 37°C. Following this incubation period, the contents of the wells were aspirated. Each well was washed twice with sterile deionized water, and the cells were resuspended in 100 μL of MHB. The biofilms were thoroughly scraped, and the total number of viable cells was quantified by serial dilution and plating on blood agar plates for bacterial cultures or Sabouraud’s dextrose agar for *C. albicans* to determine CFU. MBEC_90_ values were established as the lowest concentrations of NaOCl, resulting in a 90% reduction in cell viability within the biofilms relative to the untreated control.

### 2.8 Metabolic activity of biofilm cells

The metabolic activity of planktonic and biofilm isolates was determined by a resazurin-based assay for bacterial cells or 2,3-bis(2-methoxy-4-nitro-5-sulfophenyl) -5-[(phenylamino) carbonyl]-2H- tetrazolium hydroxide (XTT) reduction assay for yeast cells as described previously (Di Domenico et al., 2018; Sivori et al., 2022). All experiments were conducted in triplicate and repeated independently at least three times.

### 2.9 eDNA quantification in biofilm

For biofilms that had already formed, eDNA quantification was carried out by first adding 100 μL of TE buffer to each well, followed by 100 μL of a freshly prepared PicoGreen solution (consisting of 1 μL PicoGreen dye diluted in 199 μL TE buffer). The wells containing PicoGreen were then incubated for 5 min. Subsequently, fluorescence intensity was measured using a Wallace Victor 3, 1,420 Multicolor fluorescence plate reader (PerkinElmer), with settings at excitation 485 nm and emission 535 nm, and a 0.1-s integration time. This procedure was replicated across three biological replicates for each strain. A standard curve was generated using Lambda DNA (Invitrogen Molecular Probes) for each assay run (Sivori et al., 2022).

### 2.10 Biofilm imaging

Biofilms were grown on μ-Slide (Ibidi, Gräfelfing, Germany) inoculated with ~1 × 10^5^ cells in 500 μL of fresh MH medium and incubated for 48 h at 37°C. The culture medium was changed after 24 h of biofilm growth. Then, biofilms were treated with different concentrations of NaOCl in a fresh MH medium and incubated for an additional 22 h at 37°C. According to supplier specifications, biofilms were stained using the LIVE/DEAD BacLight Bacterial Viability Kit (Life Technologies, New York, NY, United States) and examined with an Axio Observer inverted fluorescence microscope equipped with an Apotome system (Carl Zeiss International, Oberkochen, Germany). Data was analyzed using ZEN 3.2 (blue edition) software (Truglio et al., 2024). All the images are available in Supplementary Figure S1.

### 2.11 Statistical analysis

We reported the categorical variables through absolute and relative frequencies, whereas the continuous variables through median and means with standard deviations (SD). Kolmogorov–Smirnov normality test was calculated for all the continuous variables and the Kruskal-Wallis test was used to evaluate the differences between groups. Since we are dealing with multiple comparisons, the Bonferroni correction was applied. The correlation between variables was investigated using the Spearman correlation coefficient. A univariate regression linear analysis was conducted to investigate the relationship between variables. Statistical analyses were performed with GraphPad Prism 10.0 (GraphPad Software, Inc., San Diego, CA, United States). Differences were considered statistically significant for *p*-values of *p* < 0.05 (*), *p* < 0.01 (**), and *p* < 0.001 (***).

## 3 Results

**Figure 1:**
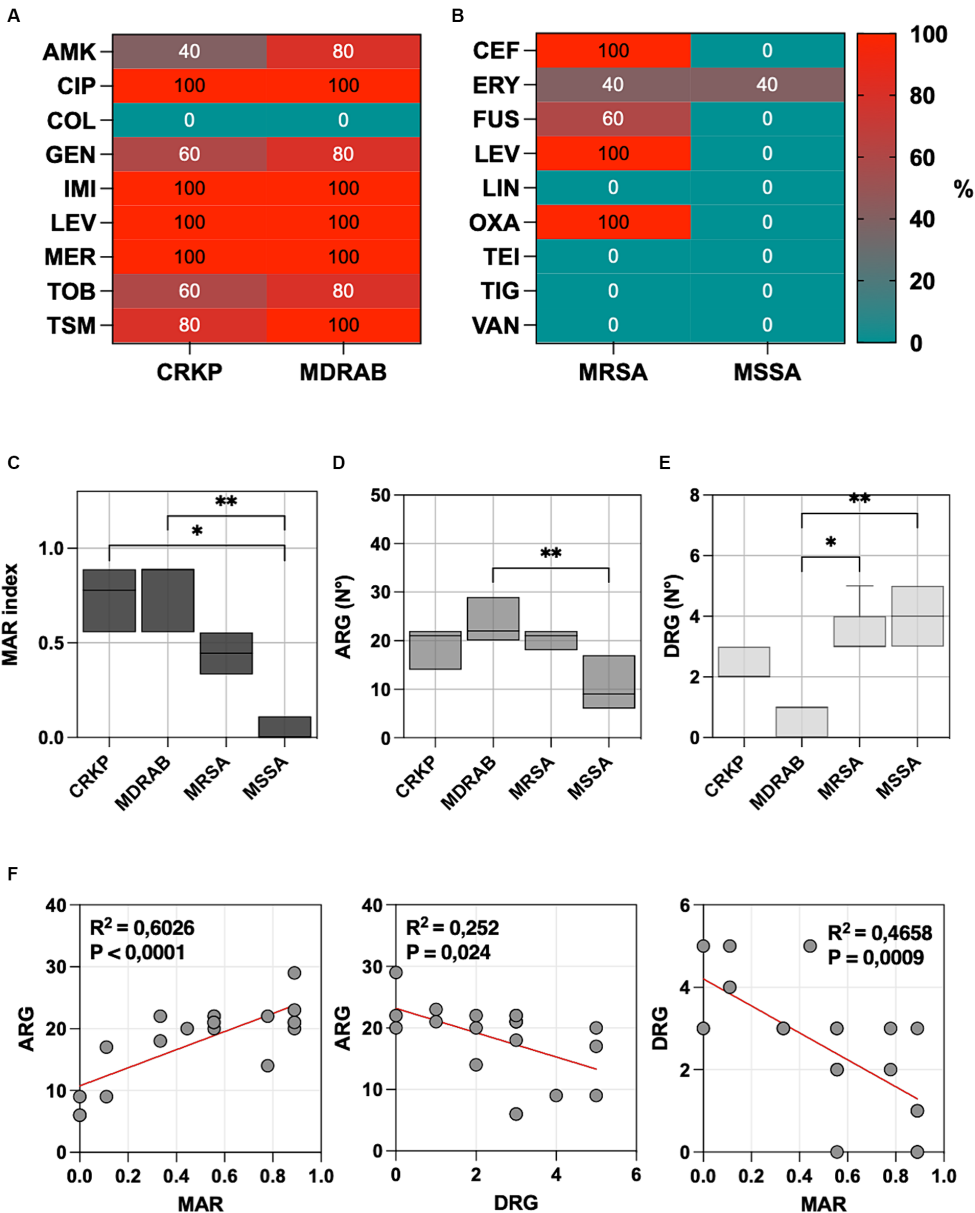
**(A)** The percentage of carbapenem-resistant *Klebsiella pneumoniae* (CRKP), multidrug-resistant *Acinetobacter baumannii* (MDRAB), tested non-susceptible or resistant to amikacin (AMK), ciprofloxacin (CIP), colistin (COL), gentamicin (GEN), imipenem (IMI), levofloxacin (LEV), meropenem (MER), tobramycin (TOB) and trimethoprim-sulfamethoxazole (TSM). **(B)** The percentage of methicillin-resistant *Staphylococcus aureus* (MRSA) and methicillin-susceptible *S. aureus* (MSSA) tested non-susceptible or resistant to ceftaroline (CEF), daptomycin (DAP), erythromycin (ERY), fusidic acid (FUS), levofloxacin (LEV), linezolid (LIN), oxacillin (OXA), teicoplanin (TEI), tigecycline (TIG), and vancomycin (VAN). **(C)** Multiple antibiotic resistance (MAR) index box plots, **(D)** predictions of antibiotic resistance genes (ARG), and **(E)** disinfectant resistance genes (DRG) using CARD v3.2.8. **(F)** Linear regression analysis illustrates the correlation among the MAR index, ARGs, and DRGs. Significance was assessed by the Kruskal Wallis statistic test. ^*^, *p* < 0.05; ^**^, *p* < 0.01; ^***^, *p* < 0.001, ^****^, *p* < 0.0001.

Bacterial isolates comprised five carbapenem-resistant *Klebsiella pneumoniae* (CRKP), five multidrug-resistant *Acinetobacter baumannii* (MDRAB), five methicillin-resistant *Staphylococcus aureus* (MRSA), and five methicillin-susceptible *Staphylococcus aureus* (MSSA). Antibiotic susceptibility patterns are summarized in Figures 1A,B.

All CRKP and MDRAB isolates were resistant to fluoroquinolones (ciprofloxacin and levofloxacin) and carbapenems (imipenem and meropenem), with colistin being the sole effective agent. MRSA displayed resistance to ceftaroline, levofloxacin, and oxacillin, while MSSA showed a generally lower resistance profile, with an 80% resistance noted only to erythromycin. The MIC values for each strain and the different antibiotics are provided in Supplementary Table S1.

The Multiple Antibiotic Resistance (MAR) index for each isolate was determined by dividing the number of antibiotics to which the isolate was resistant by the total number of antibiotics tested (Figure 1C). The highest MAR index was observed in MDRAB (median 0.889, range 0.556–0.889), followed by CRKP (median 0.778, range 0.556–0.889), and MRSA (median 0.444, range 0.333–0.556). MSSA isolates (median 0.000, range 0.000–0.111) had a significantly lower MAR index compared to MDRAB (*p* = 0.001) and CRKP (*p* = 0.014), but not to MRSA.

Whole-genome sequencing (WGS) identified three CRKP isolates as sequence type (ST) 307 and two as ST501. In MDRAB, four isolates were classified as ST2 and one as ST139. MRSA predominantly belonged to ST22 (n = 4), with a single ST5 isolate. MSSA demonstrated greater diversity, with ST398 being the most frequent (n = 3), followed by single isolates of ST5 and ST7.

All CRKP strains harbored the SHV β-lactamase genes responsible for extended-spectrum β-lactamases (ESBLs). Specifically, SHV-11 and SHV-28 were present in two strains, while SHV-1 was detected in one strain. Additionally, two strains possessed the OXA-1 gene, contributing to ESBL resistance. Notably, all the CRKP strains tested positive for the carbapenem-hydrolyzing β-lactamase KPC gene. In one positive isolate for SHV, OXA-1, and KPC, the ompK37 gene, which confers reduced permeability to β-lactams, was also identified. Furthermore, all CRKP isolates harbored 4 to 6 quinolone resistance genes.

In MDRAB, class D oxacillinase (OXA) resistance genes were the most commonly identified. Three isolates were found to have OXA-23. All isolates also carried other OXA genes, such as two OXA66, two OXA82, and one OXA545. Significantly, the MDRAB strains were positive for the multidrug efflux pump genes adeIJK.

All MRSA isolates resistant to levofloxacin carried the gyrA and parC genes, which are known to confer resistance to quinolones. As expected, the mecA gene conferring resistance to oxacillin was detected in all MRSA isolates. In MSSA, erythromycin ribosome methylase genes (ermA or ermC) were identified in 2 isolates.

Predictions of antibiotic resistance genes (ARGs) and disinfectant resistance genes (DRGs) showed that MDRAB exhibited the highest level of ARGs, with a median number of 22 genes (range 20–29), followed by CRKP (21 genes, range 14–22), MRSA (21 genes, range 18–22), and MSSA (9 genes, range 6–17), as reported in Figure 1D. Conversely, DRGs were more abundant in MSSA (4 genes, range 3–5), followed by MRSA (3 genes, range 3–5), CRKP (2 genes, range 2–3), and MDRAB (0 genes, range 0–1), as indicated in Figure 1E.

All CRKP isolates contain kpnEF genes, which confer resistance to broad-spectrum disinfectants. The qacEΔ1 gene, linked to antiseptic resistance, appears in 40% of CRKP and MDRAB. In *S. aureus*, the NorC efflux pump, crucial for resistance to various disinfectants, is universally present. MRSA showed a median of 3 genes related to resistance in disinfectant. In particular, all the isolates were positive for arlR and mgrA, which modulates the expression of norA and norB. Similarly, MgrA regulates the expression of a multitude of genes, including the regulation of several multidrug efflux pumps, including NorA, NorB, and NorC. The other efflux pump linked to resistance to disinfectant is SdrM, which has been detected in 3 MSSA and one MRSA strain.

Linear regression analysis (Figure 1F), plotting the number of MAR index and ARG, showed a positive correlation (*p* < 0.0001). Conversely, a negative correlation was observed between ARG and DRG (*p* = 0.0240) and DRG and AMR (*p* = 0.0009).

### 3.1 Comparative analysis of biofilm production in clinical bacterial pathogens

WGS analysis revealed the widespread occurrence of genes responsible for adhesion and biofilm formation. In particular, CRKP harbored the mrkABCDF gene cluster, encoding type 3 fimbriae mediating adhesion to abiotic surfaces and biofilm formation and crucial for persistence in the nosocomial setting.

MDRAB isolates consistently harbored genes for producing pili and other surface structures that facilitate adherence and biofilm development. Genes such as bap for biofilm-associated protein and csuABCDE for chaperone-usher pili assembly were detected in 83.3% of MDRAB isolates, indicating a common biofilm-forming strategy.

In *S. aureus*, both MSSA and MRSA strains showed the presence of the icaADBC operon, which is instrumental in intercellular adhesion and biofilm polysaccharide synthesis. Additionally, fibronectin-binding protein genes fnbA and fnbB were ubiquitously present, indicating a potential mechanism for cellular adhesion in diverse environmental niches. Clumping factor genes clfA and clfB, along with the collagen adhesion gene cna and the biofilm-associated sasG, were also identified, highlighting the multifaceted approach of *S. aureus* in establishing infection and chronicity.

**Figure 2:**
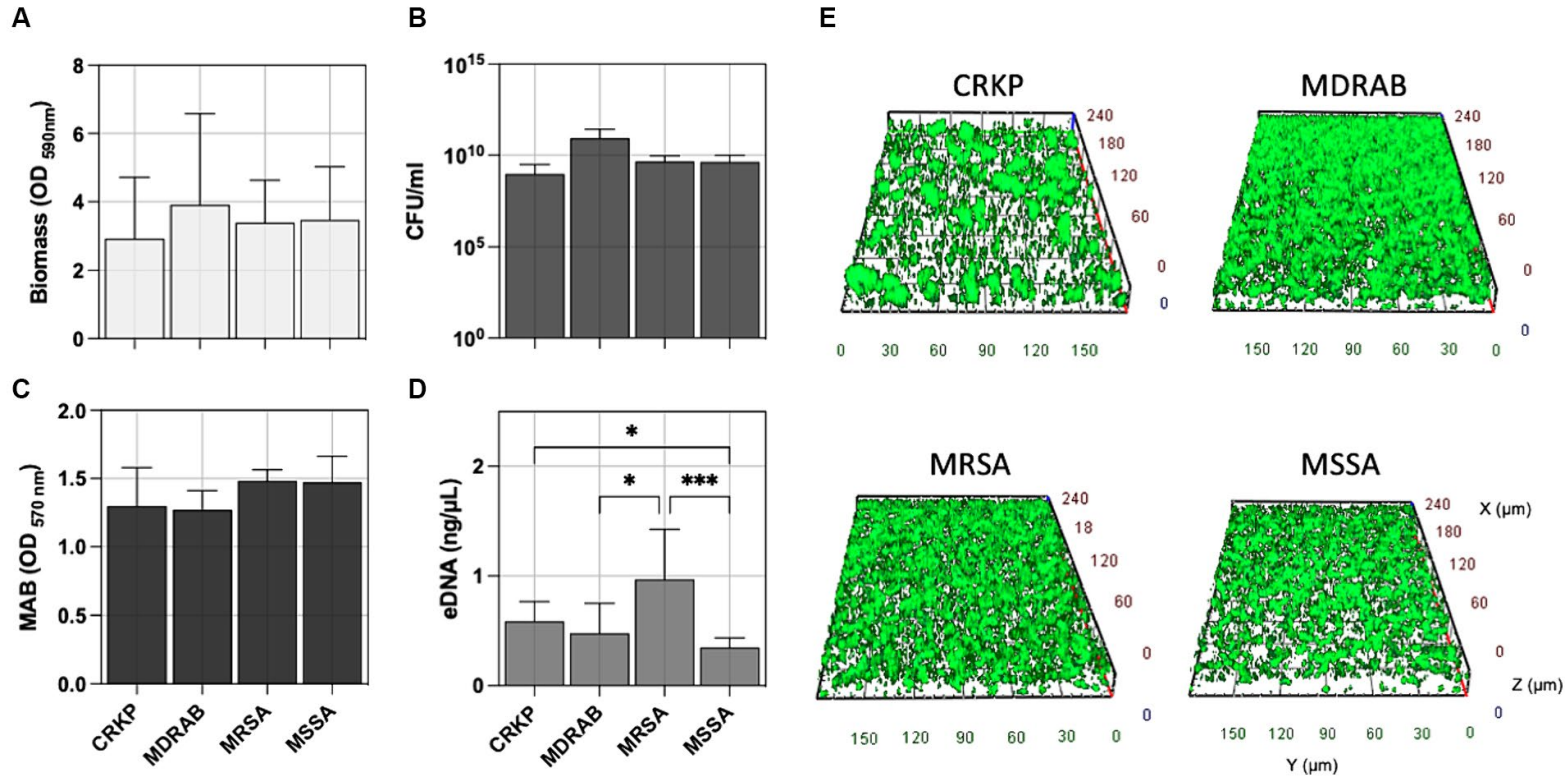
Comparative biofilm analysis of carbapenem-resistant *Klebsiella pneumoniae* (CRKP), multidrug-resistant *Acinetobacter baumannii* (MDRAB), methicillin-resistant *Staphylococcus aureus* (MRSA), and methicillin-susceptible *S. aureus* (MSSA) was quantitatively assessed by evaluating **(A)** the biomass using crystal violet (CV) staining, **(B)** the count of biofilm-forming cells (CFU/ml), **(C)** metabolic activity of biofilm cell (MAB) expressed as the area under the growth curve by the resazurin assay, and **(D)** the amount of extracellular DNA (eDNA) in the biofilm matrix. **(E)** Representative fluorescence microscopy images of biofilm formation for the indicated bacterial species stained with the Live/Dead BacLight kit after 24 h of incubation at 37°C. Computerized 3D reconstruction (X, Y, and Z axis) of the biofilm layers (in μm). Significance was assessed by the Kruskal Wallis statistic test. ^*^, *p* < 0.05; ^**^, *p* < 0.01; ^***^, *p* < 0.001, ^****^, *p* < 0.0001.

Accordingly, CRKP, MDRAB, and *S. aureus* (both MSSA and MRSA) exhibited comparable levels of biofilm formation, the total number of biofilm cells expressed as CFU/ml, and the metabolic activity of the biofilm cells (MAB) as reported in Figures 2A–C. Interestingly, the amount of extracellular DNA (eDNA) within the biofilm matrix (Figure 2D) was significantly higher in MRSA isolates compared to CRKP (*p* = 0.0395), MDRAB (*p* = 0.0302), and MSSA (*p* = 0.0005). Fluorescence microscopy revealed that all isolates formed biofilms with thicknesses ranging from 25 to 45 μm, displaying typical maturation features such as cell aggregates and water channels (Figure 2E and Supplementary Figure S1).

#### 3.1.1 Efficacy of sodium hypochlorite against planktonic and biofilm-forming microbial isolates

**Figure 3:**
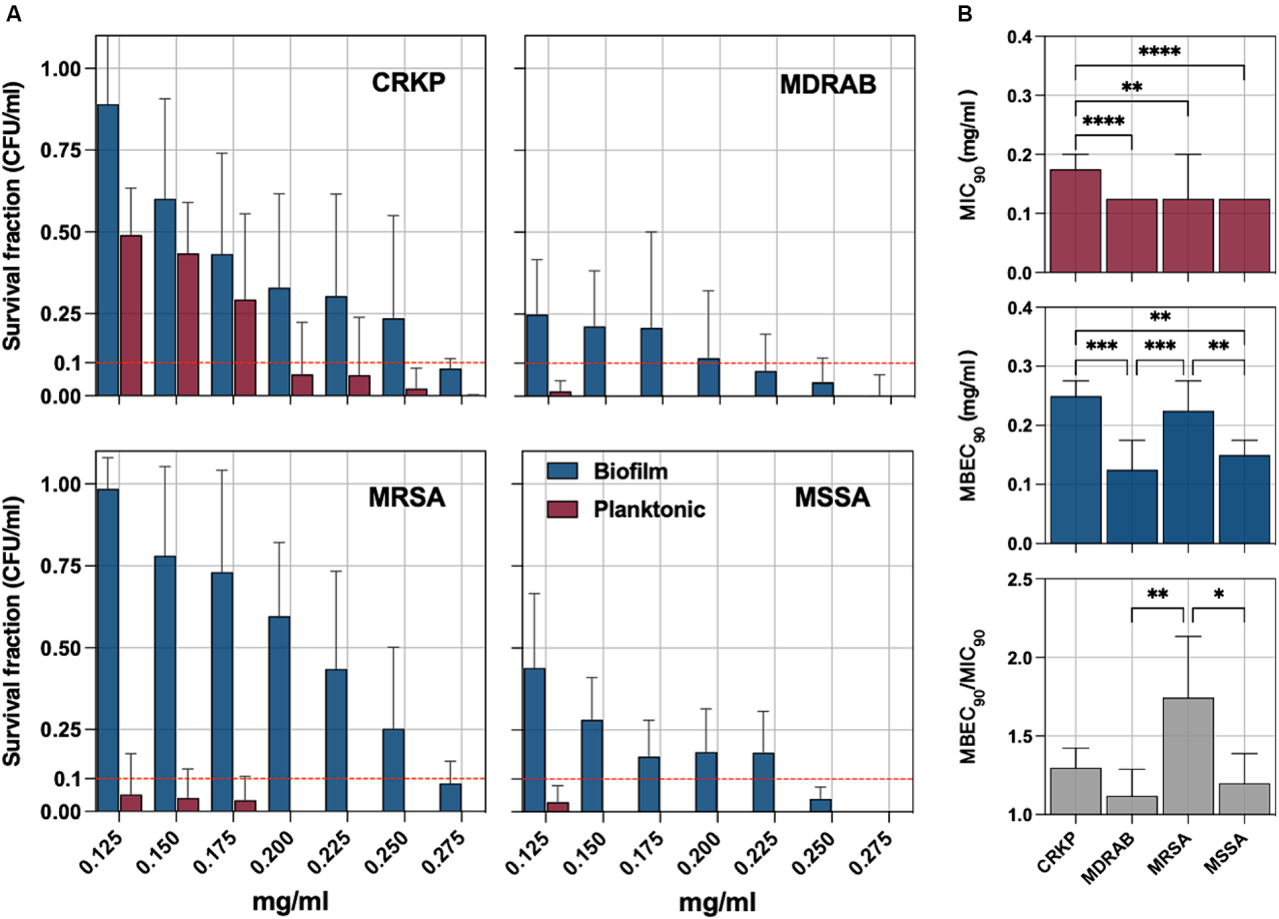
**(A)** The antimicrobial and antibiofilm activity of an electrolytic sodium hypochlorite solution (NaOCl) against carbapenem-resistant *Klebsiella pneumoniae* (CRKP), multidrug-resistant *Acinetobacter baumannii* (MDRAB), methicillin-resistant *Staphylococcus aureus* (MRSA), and methicillin-susceptible *S. aureus* (MSSA) is displayed through survival cell fractions compared to untreated control strains at concentrations ranging from 0.125 to 0.275 mg/mL. **(B)** The minimum inhibitory concentration (MIC_90_) is the lowest concentration (mg/ml) needed to inhibit 90% of planktonic bacterial growth relative to controls and the minimum biofilm eradication concentration (MBEC_90_). The antimicrobial and antibiofilm activity of NaOCl is demonstrated through the survival of bacterial cell fractions, compared to untreated control strains, at concentrations ranging from 0.125 to 0.275 mg/mL. The minimum inhibitory concentration (MIC_90_) and the minimum biofilm eradication concentration (MBEC_90_) are defined as the lowest NaOCl concentrations required to inhibit 90% of planktonic and biofilm bacterial growth, respectively, compared to untreated controls. The MBEC_90_/MIC_90_ ratio was used to quantify the biofilm tolerance to NaOCl for all tested strains. Significance was assessed by the Kruskal Wallis statistic test. ^*^, *p* < 0.05; ^**^, *p* < 0.01; ^***^, *p* < 0.001, ^****^, *p* < 0.0001.

The bacterial pathogens were exposed to sodium hypochlorite (NaOCl) to evaluate its antimicrobial and antibiofilm efficacy. Survival fractions of cells were assessed in comparison to untreated control strains, with NaOCl concentrations ranging from 0.125 to 0.275 mg/mL (Figure 3A). The minimum inhibitory concentration (MIC_90_), defined as the lowest concentration needed to inhibit 90% of planktonic bacterial growth relative to controls, was determined. For planktonic isolates, the MIC_90_ was 0.175 mg/mL for CRKP and 0.125 mg/mL for MDRAB, MRSA, and MSSA (Figure 3B). Biofilm-forming isolates were further tested to determine the minimum biofilm eradication concentration (MBEC_90_), which is the lowest concentration of NaOCl needed to eradicate 90% of the biofilm biomass. The MBEC90 values were found to be 0.250 mg/mL for CRKP, 0.125 mg/mL for MDRAB, 0.225 mg/mL for MRSA, and 0.150 mg/mL for MSSA (Figure 3B). Overall, the MBEC_90_ values were significantly higher (*p* < 0.001) than the corresponding MIC_90_ values, indicating a greater resistance of biofilm-associated cells to NaOCl treatment.

The MBEC_90_/MIC_90_ ratio, indicating the fold increase in the NaOCl dose needed to inhibit or kill cells in biofilms compared to planktonic growth, was used to quantify biofilm tolerance. The MBEC_90_/MIC_90_ ratio was significantly higher in the MRSA group compared to the MSSA (*p* = 0,0286) and MDRAB (*p* = 0,0014) but not CRKP (Figure 3B).

#### 3.1.2 Determination of NaOCl activity against *Candida albicans* isolates

**Figure 4:**
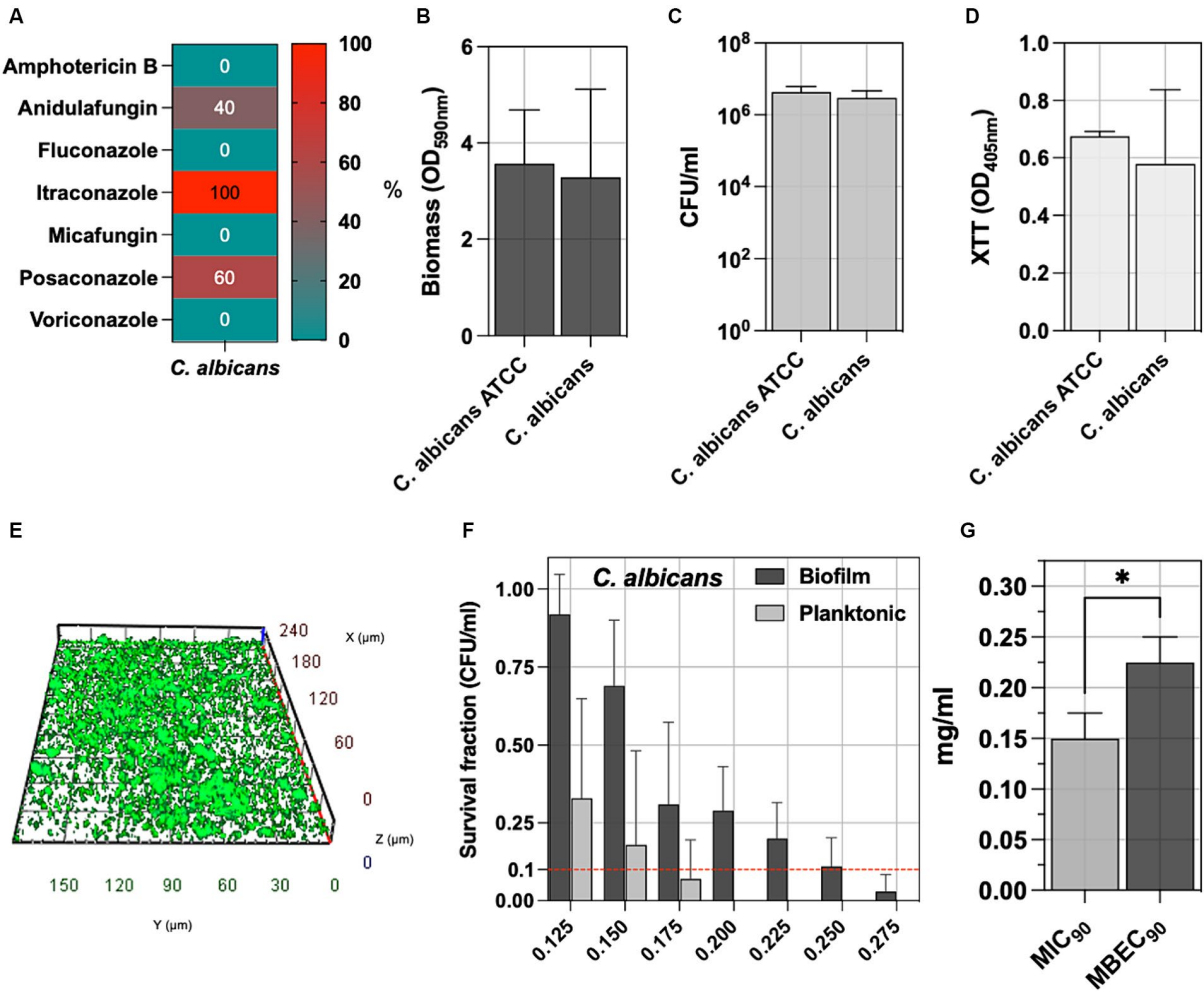
**(A)** The percentage of *C. albicans* tested non-susceptible or resistant according to EUCAST breakpoints to amphotericin B, anidulafungin, fluconazole, itraconazole, micafungin, posaconazole, voriconazole. **(B)** The biofilm of 5 *C. albicans* clinical isolates was assessed by measuring the biomass with CV staining, **(C)** the count of biofilm-forming cells (CFU/ml), and **(D)** the metabolic activity of the biofilm cells with XTT. **(E)** Representative fluorescence microscopy image stained with the Live/Dead BacLight kit after 24 h of incubation at 37°C. The *C. albicans* ATCC 2091, classified as a strong biofilm producer, was included as a control strain. **(F)** The antimicrobial and antibiofilm activity of NaOCl against *C. albicans* isolates was demonstrated through survival cell fractions, compared to untreated control strains, at concentrations ranging from 0.125 to 0.275 mg/mL. **(G)** The minimum inhibitory concentration (MIC_90_) and the minimum biofilm eradication concentration (MBEC_90_) are defined as the lowest NaOCl concentrations (mg/ml) required to inhibit 90% of planktonic and biofilm bacterial growth, respectively, compared to untreated controls. Significance was assessed by the Kruskal Wallis statistic test. ^*^, *p* < 0.05; ^**^, *p* < 0.01; ^***^, *p* < 0.001, ^****^, *p* < 0.0001.

*C. albicans* is the main fungal species involved in pressure ulcer infection; accordingly, five isolates were included in the study. Figure 4A reports the antifungal susceptibility testing (AfST) data for the *C. albicans* clinical isolates. According to EUCAST clinical breakpoints, all the isolates were susceptible to amphotericin B, fluconazole, micafungin, and voriconazole. Resistance was observed in anidulafungin (40%), posaconazole (60%) and itraconazole (100%).

Biofilm was assessed by measuring biomass production, counting the total number of biofilm cells expressed as CFU/ml, determining the metabolic activity of the biofilm cells (XTT), and comparing it with the reference *C. albicans* ATCC 2091 strain (Figures 4B–D). No significant differences were observed between *C. albicans* clinical isolates and the *C. albicans* ATCC 2091, classified as a strong biofilm producer strain (Di Domenico et al., 2018). Fluorescence microscopy reveals a well-defined architecture within the *C. albicans* biofilm, as evidenced by the consistent thickness between 20 to 35 μm. The three-dimensional analysis further highlights the presence of dense cellular clusters and heterogeneity of the biofilm matrix, underscoring the spatial organization (Figure 4E and Supplementary Figure S1).

The antifungal activity of NaOCl against planktonic and biofilm yeast cells, ranging from 0.125 to 0.275 mg/mL, was tested against *C. albicans* isolates (Figure 4F). Cell survival fractions were assessed in comparison to untreated control strains. NaOCl showed an MIC_90_ of 0.150 mg/mL (0.125–0.175 mg/mL) against planktonic *C. albicans*, while the MBEC_90_ was 0.225 mg/mL (0.175–0.250 mg/mL) (Figure 4G). The MBEC_90_/MIC_90_ ratio showed a median value of 1.43 (range 1.29–1.80) in the biofilm-mediated tolerance in *C. albicans* isolates (Figure 3H).

## 4 Discussion

This study provides valuable insights into the *in vitro* efficacy of a NaOCl solution against clinical bacterial pathogens and *C. albicans* isolated from patients with PUs. The presence of strains such as CRKP, MDRAB, MRSA, and MSSA within PUs mirrors the findings of previous studies, which have similarly noted these pathogens as significant contributors to the complexity of wound infections (Li et al., 2021). The high levels of AMR observed in MDRAB and CRKP are alarming, given their role in healthcare-associated infections and the growing prevalence in PUs worldwide (Zhen et al., 2020). Similarly, MRSA presents significant concerns in managing PU infections due to its multidrug resistance profile correlated with suboptimal wound healing outcomes and extended durations of hospitalization (Binsuwaidan et al., 2023). In the current study, a MAR index greater than 0.4 indicates a high prevalence of multiple drug resistance among clinical isolates (Krumperman, 1983; Jain et al., 2021). The MAR index has been confirmed as an effective metric for assessing the risk associated with microbial isolates. Indeed, linear regression analysis delineates a statistically significant positive correlation between the MAR index and the number of ARGs. This relationship reinforces the utility of the MAR index not only as an essential epidemiological tool to monitor drug resistance but also as a predictor of the genetic propensity for resistance within bacterial populations in PUs (Krumperman, 1983; Jain et al., 2021).

WGS has refined our understanding of bacterial strains implicated in PU infections, revealing high-risk clones such as CRKP ST307 and ST501, which are known for their adaptability and prevalence in healthcare settings (Villa et al., 2017; Fasciana et al., 2019; Heiden et al., 2020). In MDRAB, four isolates were classified as ST2, the globally predominant ST. This ubiquity poses a significant challenge to infection control, underscoring the need for a concerted global response to effectively manage its spread (Cherubini et al., 2022). The ST22 was the most abundant detected in MRSA strains in four isolates. ST22 is particularly concerning due to its association with virulence factors such as Panton-Valentine leucocidin and toxic shock syndrome toxin 1 genes, which may enhance the ability to cause severe infections (Kaneko et al., 2023). Conversely, MSSA demonstrated a greater sequence type diversity than their Gram-negative counterparts and MRSA, with ST398 being the most frequent. The diversity observed in MSSA could reflect a broader range of ecological niches and host interactions, potentially impacting their pathogenicity and transmission dynamics (Vandendriessche et al., 2014).

In these highly virulent STs, the genetic determinants of resistance to frontline antibiotics underscore the pathogens’ capacity to withstand conventional antibiotic therapy and the need for alternative treatment strategies. Accordingly, topical antiseptics, including NaOCl, are favored over antibiotics due to rising drug resistance and are recommended as first-line treatment for infected skin lesions (McCullough and Carlson, 2014). Specifically, NaOCl has been demonstrated to be active against biofilms by denaturing proteins in the matrix and inhibiting major enzymatic functions in bacterial cells. Although NaOCl disinfectants are effective against biofilms at concentrations lower than those typically found in commercial products, the use of sub-lethal concentrations of some sodium-containing disinfectants could promote the formation of biofilms on environmental surfaces (Barnes and Greive, 2013; Cincarova et al., 2016; Lineback et al., 2018).

Our study demonstrated that CRKP exhibited significantly higher MIC_90_ values for NaOCl than the other isolates. For CRKP, the higher MIC_90_ and MBEC_90_ values compared to the other pathogens could be partially explained by the presence of kpnEF genes, which are involved in broad-spectrum antimicrobial resistance and clinically relevant disinfectants (Srinivasan and Rajamohan, 2013). In addition, two of the CRKP strains also have the qacEΔ1 gene that confers multiple resistance to antibiotics and antiseptics in Gram-negative bacteria (Kucken et al., 2000). The diminished efficacy of NaOCl may result from the activity of these efflux pumps actively removing disinfectants from the cell, thereby reducing the disinfectant’s ability to achieve the desired antimicrobial effect. This would require higher concentrations of NaOCl to inhibit or eradicate CRKP, as reflected in the MIC_90_ and MBEC_90_ values. MDRAB exhibited a low frequency of genes related to disinfectants. In particular, two isolates have the qacEΔ1 gene and one the amvA gene (Rajamohan et al., 2010; Short et al., 2021). Accordingly, MDRAB showed a lower MIC_90_ and MBEC_90_ to CRKP despite exhibiting a comparable biomass level.

For MRSA, the presence of regulatory systems like ArlRS and MgrA and efflux pumps like NorC may indicate a sophisticated system of resistance that operates beyond just preventing the entrance of antiseptics and could involve regulatory pathways and interactions with the cell’s stress responses (Liang et al., 2005; Truong-Bolduc et al., 2005; Costa et al., 2024).

The observed MBEC_90_ values for MSSA, which are lower than those for MRSA, imply a differential efficacy of the MSSA efflux pump system in the presence of NaOCl or point to inherent structural or compositional variations in MSSA biofilms that may render them more susceptible to NaOCl-mediated disinfection (McCarthy et al., 2015; Oliva et al., 2021). Indeed, in MSSA, the most prevalent efflux pump linked to resistance to disinfectant is SdrM, which shares 23% identity with the *S. aureus* MDR efflux pumps NorB (Yamada et al., 2006). Additionally, the biofilm matrix composition, particularly the eDNA content, could account for variations in disinfection susceptibility. The significantly higher eDNA content in MRSA biofilms compared to MSSA might confer a greater structural integrity or protection against NaOCl, considering that eDNA is a known contributor to biofilm stability and antimicrobial resistance (McCarthy et al., 2015; Sivori et al., 2022). Hence, while MSSA and MRSA biofilms demonstrate similar thicknesses and morphological maturation, the divergent eDNA concentrations suggest a structural disparity with potential implications for disinfection strategies.

Indeed, our findings demonstrated that the MBEC_90_/MIC_90_ ratio was significantly higher in the MRSA group compared to the MSSA and MDRAB groups but not when compared to CRKP. This observation suggests the possible contribution of specific elements within the biofilm matrix to the increased tolerance observed. Notably, matrix characterization revealed that MRSA strains have a higher level of eDNA content within their biofilm matrix. This higher eDNA content may play a crucial role in limiting the activity of NaOCl, as eDNA can contribute to the structural integrity and protective function of the biofilm, potentially impeding the penetration and efficacy of antimicrobial agents like NaOCl (Sivori et al., 2022). Further studies are warranted to elucidate the exact mechanisms by which eDNA influences biofilm resilience and to explore potential strategies to counteract this protective effect.

The efficacy of NaOCl against both planktonic and biofilm-forming microbial isolates, as reported in this study, highlights its significant antimicrobial and antibiofilm potential. Notably, the MIC_90_ values, ranging from 0.125 mg/mL for MDRAB, MRSA, and MSSA to 0.175 mg/mL for CRKP, underline the effectiveness of NaOCl in inhibiting bacterial growth at concentrations considerably lower than those typically found in commercial disinfectants. These findings are consistent with prior studies indicating that sodium hypochlorite disinfectants can effectively reduce biofilms (DeQueiroz and Day, 2007; Tiwari et al., 2018; Koti et al., 2024). This aligns with previous findings that noted that NaOCl and hydrogen peroxide disinfectants destroy both the biofilm matrix and the bacteria cells within, establishing them as superior anti-biofilm agents (DeQueiroz and Day, 2007; Lineback et al., 2018; Tiwari et al., 2018). Nevertheless, the MBEC_90_ values are markedly higher than MIC_90_, suggesting that NaOCl requires increased concentrations while effectively disrupting established biofilms. This observation is also consistent with past research, suggesting that biofilms pose significant challenges to disinfectant efficacy due to their dense cellular matrices and protective barriers (Gilbert et al., 2001; Mitchell et al., 2015).

A previous study raises critical points about the potential for sub-lethal concentrations of sodium-containing disinfectants to inadvertently promote biofilm formation (Cincarova et al., 2016). This phenomenon represents a paradoxical risk, where disinfectant use intended to control pathogen spread instead enhances their protective niches. Specifically, our quantification of biofilm cells (CFU/ml) did not indicate increased biofilm production for our isolates under sub-lethal concentrations of NaOCl. However, we cannot exclude the possibility of increased biomass production. Indeed, our study’s focus on measuring the bactericidal effects via cell counts offers a direct evaluation of antibacterial activity rather than biomass estimation alone.

*C. albicans*, showed susceptibility to amphotericin B, fluconazole, micafungin, and voriconazole, confirming their efficacy as initial treatments in clinical settings. Conversely, resistance to anidulafungin, posaconazole, and itraconazole raises concerns about emerging antifungal resistance (Kessler et al., 2022). NaOCl displayed differential efficacy against planktonic and biofilm forms of ***C. albicans***. While NaOCl effectively inhibits planktonic cells at low concentrations, higher concentrations are needed to eradicate biofilms, as evidenced by higher MBEC_**90**_ compared to MIC_**90**_ values, reflecting biofilms’ inherent resistance (Short et al., 2021). Importantly, the consistency in biofilm structure across the clinical isolates and the reference strain indicates that NaOCl must overcome a common, robust defense mechanism to be effective. The concentration of NaOCl required for biofilm eradication implies that it could play a role in disinfection protocols aimed at preventing the spread of *C. albicans*, particularly in healthcare environments. This positions NaOCl as a potentially valuable agent in the disinfection arsenal against *C. albicans* in both planktonic and biofilm states as confirmed by the low MBEC_90_/MIC_90_ ratio.

This study provides valuable insights but has limitations affecting its generalizability. The modest sample size of 25 isolates may not fully reflect the diversity of resistance patterns or biofilm capabilities seen in broader clinical contexts. Additionally, while WGS offers detailed genetic perspectives on resistance mechanisms, the functional impact on clinical outcomes is still speculative. The findings on biofilm susceptibility to NaOCl, derived from *in vitro* single-species cultures, might not accurately mimic the complex conditions in PU infections. These factors underscore the necessity for extended research with larger and more varied samples and more comprehensive antimicrobial evaluations to bridge the gap between laboratory and clinical settings. Overall, while these limitations highlight the need for further research, the study demonstrates that the efficacy of NaOCl against both planktonic and biofilm-forming microbial isolates occurs at concentrations significantly lower than the 0.5 mg/mL typically found in commercial disinfectant products (Maniscalco et al., 2023). This indicates that even at diluted concentrations, NaOCl is effective in targeting and controlling these clinically relevant microbes, reinforcing its valuable role in infection control and disinfection protocols.

## 5 Conclusion

The application of NaOCl demonstrated a potent antimicrobial and antibiofilm activity, though it was markedly more effective against planktonic than biofilm-embedded cells. The low MBEC_90_/MIC_90_ ratio suggests that the biofilm matrix is poorly effective in protecting the bacterial and *C. albicans* isolates from NaOCl.

The observed susceptibility of bacterial pathogens and *C. albicans* to NaOCl, both in planktonic and biofilm states, suggests that NaOCl could be a broad-spectrum agent applicable in a multi-pathogen context, reducing the microbial burden and promoting PU’s healing (Serena et al., 2022). This distinction between MIC_90_ and MBEC_90_ values points to the resilience of biofilm architectures and emphasizes the need for higher concentrations of NaOCl for biofilm eradication of microbial isolates from PUs.

Interestingly, the correlation analyses reveal a nuanced interaction in CRKP and MRDAB between biofilm biomass, cell count, and DRGs, which may inform future studies aiming to disrupt biofilm formation or enhance disinfectant penetration in the biofilm matrix.

Ultimately, our study supports the use of electrolytic NaOCl solutions as a potential antiseptic at concentrations considerably lower than those typically found in commercial disinfectants.

## Data availability statement

The original contributions presented in the study are included in the article/Supplementary material, further inquiries can be directed to the corresponding author.

## Author contributions

GF: Writing – original draft, Writing – review & editing. FS: Writing – original draft, Writing – review & editing. IC: Writing – original draft, Writing – review & editing. MT: Writing – original draft, Writing – review & editing. LT: Writing – original draft, Writing – review & editing. FS: Writing – original draft, Writing – review & editing. MF: Writing – original draft, Writing – review & editing. FO: Writing – original draft, Writing – review & editing. LP: Writing – original draft, Writing – review & editing. DK: Writing – original draft, Writing – review & editing. FP: Writing – original draft, Writing – review & editing. ED: Writing – original draft, Writing – review & editing.

## Footnotes

^1^https://www.ecdc.europa.eu/en
