# Supplementary figures and images for: Efficacy of sodium hypochlorite in overcoming antimicrobial resistance and eradicating biofilms in clinical pathogens from pressure ulcers
Source: Front Microbiol. 2024 Jul 10;15:1432883. doi: 10.3389/fmicb.2024.1432883 (PMC11266179; doi:10.3389/fmicb.2024.1432883)

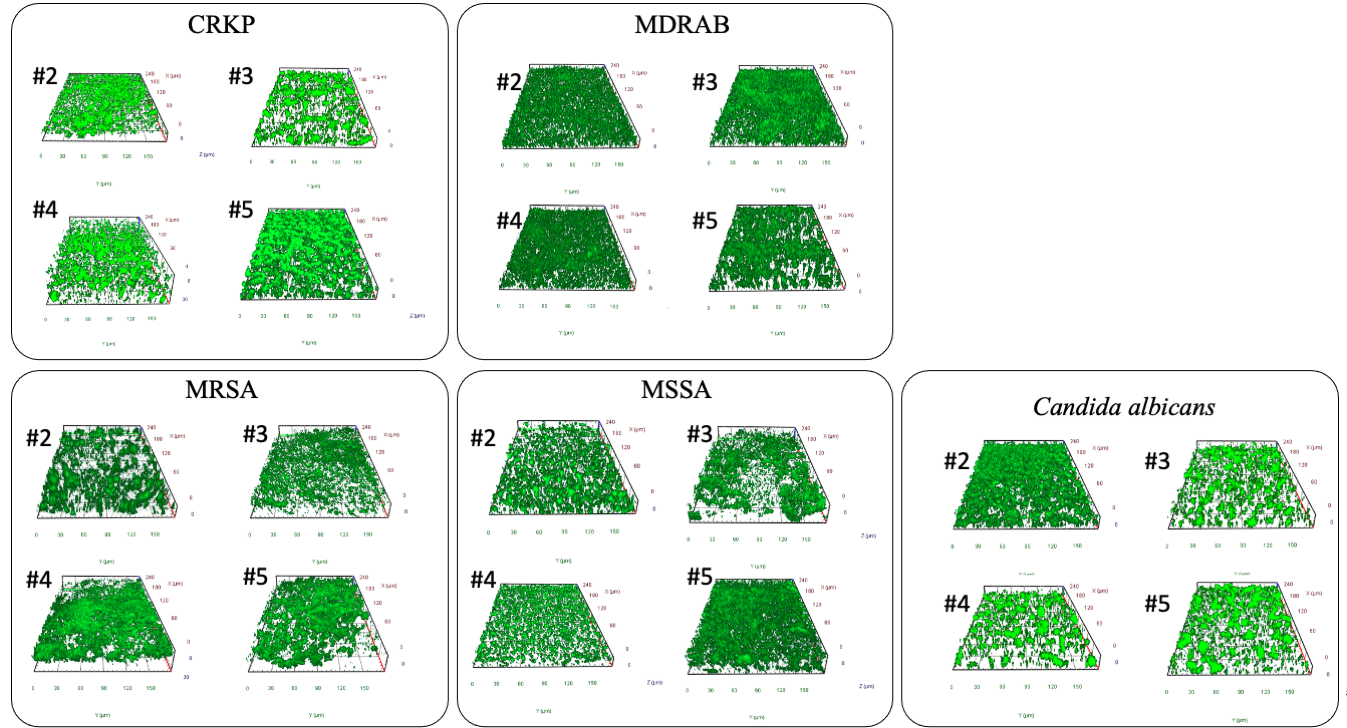

Supplement: Supplementary file 1 [file Image_1.TIFF]
